# Implication of Snail in Metabolic Stress-Induced Necrosis

**DOI:** 10.1371/journal.pone.0018000

**Published:** 2011-03-23

**Authors:** Cho Hee Kim, Hyun Min Jeon, Su Yeon Lee, Min Kyung Ju, Ji Young Moon, Hye Gyeong Park, Mi-Ae Yoo, Byung Tae Choi, Jong In Yook, Sung-Chul Lim, Song Iy Han, Ho Sung Kang

**Affiliations:** 1 Department of Molecular Biology, College of Natural Sciences, Pusan National University, Pusan, Korea; 2 Nanobiotechnology Center, Pusan National University, Pusan, Korea; 3 Division of Meridian and Structural Medicine, School of Oriental Medicine, Pusan National University, Pusan, Korea; 4 Department of Oral Pathology, Oral Cancer Research Institute, College of Dentistry, Yonsei University, Seoul, Korea; 5 Research Center for Resistant Cells, College of Medicine, Chosun University, Gwangju, Korea; 6 Department of Pathology, College of Medicine, Chosun University, Gwangju, Korea; Yale Medical School, United States of America

## Abstract

**Background:**

Necrosis, a type of cell death accompanied by the rupture of the plasma membrane, promotes tumor progression and aggressiveness by releasing the pro-inflammatory and angiogenic cytokine high mobility group box 1. It is commonly found in the core region of solid tumors due to hypoxia and glucose depletion (GD) resulting from insufficient vascularization. Thus, metabolic stress-induced necrosis has important clinical implications for tumor development; however, its regulatory mechanisms have been poorly investigated.

**Methodology/Principal Findings:**

Here, we show that the transcription factor Snail, a key regulator of epithelial-mesenchymal transition, is induced in a reactive oxygen species (ROS)-dependent manner in both two-dimensional culture of cancer cells, including A549, HepG2, and MDA-MB-231, in response to GD and the inner regions of a multicellular tumor spheroid system, an *in vitro* model of solid tumors and of human tumors. Snail short hairpin (sh) RNA inhibited metabolic stress-induced necrosis in two-dimensional cell culture and in multicellular tumor spheroid system. Snail shRNA-mediated necrosis inhibition appeared to be linked to its ability to suppress metabolic stress-induced mitochondrial ROS production, loss of mitochondrial membrane potential, and mitochondrial permeability transition, which are the primary events that trigger necrosis.

**Conclusions/Significance:**

Taken together, our findings demonstrate that Snail is implicated in metabolic stress-induced necrosis, providing a new function for Snail in tumor progression.

## Introduction

Necrosis is a type of cell death that is characterized by cell membrane rupture and that releases a cell's cytoplasmic contents into the extracellular space, causing a massive inflammatory response. Unlike tumor-suppressive apoptosis or autophagic cell death, necrosis has been implicated in tumor progression and aggressiveness by releasing a nuclear protein, high mobility group box 1 (HMGB1), that is normally involved in DNA bending and acts as a transcriptional regulator in nuclei but exerts tumor-promoting cytokine and angiogenic activities when released into the extracellular space during necrosis [1]–[6]. In addition, necrosis also increases the probability of proto-oncogenic mutations or epigenetic alterations [1]. The cells in the core region of solid tumors are usually confronted with metabolic stress from hypoxia and glucose depletion (GD) due to insufficient vascularization, a common feature of most solid tumors. During these metabolic or hypoxic stresses, tumor cells must adapt to the potentially lethal effects of metabolic constraint; otherwise, they would undergo cell death [7]–[9]. In tumors, metabolic stress-induced cell death mostly occurs through necrosis because tumor cells are defective in apoptotic and/or autophagic programs during carcinogenesis [2], [10]–[12]. In fact, necrosis is commonly found in the core region of solid tumors, is associated with poor prognosis and can impair many forms of anti-tumor treatment [7]–[9], [13]. Increased expression of HMGB1 and its receptor RAGE (receptor for advanced glycation end-products) has been observed in many types of tumors, including hepatomas and prostate cancer, and coexpression of HMGB1 and RAGE correlates with tumor invasiveness and poor clinical outcome [14], [15]. Thus, metabolic stress-induced necrosis has important clinical implications. Reactive oxygen species (ROS), Ca^2+^, and other factors are involved in the necrosis that occurs in response to DNA damage and TNF-α [12], [16]. However, the regulatory mechanism for metabolic stress-induced necrosis in tumors is poorly understood because it is generally considered as an accidental and genetically unprogrammed form of cell death.

Snail is a zinc finger transcription factor that induces the epithelial-mesenchymal transition (EMT) by directly repressing E-cadherin expression. It can be induced by many kinds of tumor-stimulating cytokines, such as transforming growth factor (TGF) β, Wnt, Notch, and hedgehog, in many human invasive carcinomas [17]–[25]. Snail is a highly unstable protein with a half-life of only 25 min because it is phosphorylated by GSK-3β at consensus motifs (i.e. Ser104/Ser107 or Ser96 within the DSG destruction motif), exported to cytosol, ubiquitinated by the E3 ubiquitin ligase β-Trcp, and degraded by proteasome [23]. Snail confers epithelial cells with migratory and invasive properties during tumor progression [17]–[19]. In primary tumors, Snail is expressed in the invasive regions of squamous, breast and hepatocellular carcinomas [25]–[27]. In addition, Snail protects cells from apoptosis induced by either withdrawal of survival factors or pro-apoptotic stimuli [28]–[32] and results in increased radioprotection *in vivo*. The resistance to gamma radiation-induced apoptosis caused by Snail is associated with the inhibition of PTEN (phosphatase and tensin homolog) phosphatase [31]. All of these activities are linked to tumor progression. Silencing of Snail by stable RNA interference in carcinoma cell lines leads to a reduction of *in vivo* tumor growth [33], and Snail possesses activities that promote mammary tumor recurrence [34].

In this study, we tried to identify the molecules that are involved in necrosis. Previously we demonstrated that GD, one of the stresses that causes metabolic stress in tumors [7]–[9], could induce necrosis and HMGB1 release into the extracellular space in cancer cell lines of different origins, including A549, HepG2, and MDA-MB-231 cells *in vitro* [35]. Herein, we show that Snail is implicated in metabolic stress-induced necrosis and Snail shRNA-mediated necrosis inhibition is linked to its ability to accelerate mitochondrial ROS production upon metabolic stress, thus providing a new function for Snail in tumor progression.

## Materials and Methods

### Cell culture and chemical treatment

A549, MCF-7, MDA-MB-231, MDA-MB-361, HepG2, HCT116, and HeLa cells were obtained from American Type Culture Collection, maintained in RPMI-1640 or DMEM containing 10% FBS, and treated with GD or chemicals as described previously [35]. To induce Snail in MCF-7 cells, the cells were transduced with a tet-inducible Snail expression vector and cultured in the absence or presence of DOX [25].

### Western blotting, HMGB1 release assay, LDH release assay, RT-PCR, and real-time PCR

Western blotting were performed as described previously using the following antibodies: Snail (polyclonal anti-Snail antiserum) [24], [25]; α-tubulin (Biogenex, CA); β-tubulin (Sigma); HMGB1 (BD Pharmingen, CA) [35]. A HMGB1 release assay was carried out as described previously [35]. LDH release was measured using the LDH Cytotoxicity Detection Kit (Roche Applied Science) according to the manufacturer's instructions. Transcript levels were assessed with reverse transcription-polymerase chain reaction (RT-PCR) and quantitative real-time PCR with primers for Snail, Slug, and GAPDH (Table S1).

### Microarray

Hybridization to microarrays was performed to screen the differentially expressed genes using Operon Human Whole 35 K Oligo chips (GenoCheck, Korea); a complete listing of the genes on this microarray is available at the following web site: http://www.genocheck.com. Data analysis was carried out using GeneSpring GX 7.3 (Agilent technologies). The values were normalized using the LOWESS algorithm.

Data deposition: The Affymetrix microarray data have been deposited in the Gene Expression Omnibus (GEO) database (GEO accession no. GSE24271).

### Copper-zinc superoxide dismutase (CuZnSOD) transfection

Plasmid pEGFP-CuZnSOD (provided by Dr. BJ Park, Pusan National Univ., Korea) that was constructed by inserting the CuZnSOD open reading frame into plasmid pEGFP (Clontech, Mountain View, CA) were transfected into A549 cells using jetPEI (Polyplus transfection); stable cells were then selected with 0.5 mg/ml Geneticin (G418, GIBCO BRL) according to the manufacturer's instructions.

### Short hairpin RNA (shRNA) interference

pSUPER-Snail shRNA was generated from two different annealed oligonucleotides (target 1, 5′-GATCCCCGCGAGCTGCAGGACTCTAATTCAAGAGATTAGAGTCCTGCAGCTCGCTTTTTA-3′ and 5′-AGCTTAAAAAGCGAGCTG CAGGACTCTAATCTCTTGAATTAGAGTCCTGCAGCTCGCGGG-3′ (Accession No. NM_005985) [28] and target 2, 5′-GATCCCCGCGAGTGGTTCTTCTGCGCTATTCAAGAGATAGCGCAGAAGAACCACTCGCTTTTTA-3′ and 5′-AGC TTAAAAAGCGAGTGG TTCTTCTGCGCTATCTCTTGAATAGCGCAGAAGAACCACT CGCGGG-3′) that were inserted into the HindIII and BglII sites of pSUPER.gfp/neo (Oligoengine Platform, Seattle, WA); the human Snail target sequence is underlined. Control shRNA was generated from annealed oligonucleotides (5′-GATCCCCAATTCTCCGAACGTGTCACGTTTCA AGAGAACGTGACACGTTCGGAGAATTTTTTTA-3′ and 5′-AGCTTAAAAAAATTCTCCGAACGTGTCACGTTCTCTTGAAACGTGACACGTTCGGAGAATTGGG-3′) [25]. All target sequences were designed and verified as specific for Snail by Blast searching against the human genome and RT-PCR/real-time PCR, respectively. The vectors pSUPER-control shRNA and pSUPER-Snail shRNA were transfected into A549, MDA-MB-231, HepG2, and MCF-7 cells using jetPEI, and stable cell line selection was performed with 1–2 mg/ml G418. Several clones were isolated after shRNA transfection in each cell type and individually characterized.

### Hoechst 33342 (HO)/propidium iodide (PI) staining, immunofluorescence, confocal microscopy, and live cell imaging

HO/PI staining was performed as described previously [35]. Intracellular H_2_O_2_, O_2_^-^ and mitochondrial ROS were detected using the 2′, 7′-dichlorofluorescein diacetate (DCFH-DA, Molecular Probes, 50 µM), dihydroethidium (HE, Molecular Probes, 10 µM), and MitoTracker Red CM-H_2_XRos (Molecular Probes, 50 nM), respectively, by fluorescence microscopy. ΔΨm was analyzed using JC-1. Cells were incubated with 5 mg/ml JC-1 (in DMSO) for 15 min at room temperature in darkness. After centrifugation (200 *g*, 5 min), cells were washed with 4°C PBS twice, resuspended in 0.5 ml PBS, and analyzed by fluorescence microscopy. ΔΨm was also detected by rhodamine 123. Cells were loaded with 5 µM rhodamine 123 for 30 min. The cells were then washed three times with PBS. Rhodamine 123 was excited at 488 nm and emission was detected at 525 nm. mPT pore opening was assessed by CoQC staining procedures according to the method of Petronilli *et al.* (1999) [36], with minor modification. Cells were loaded with 0.5 µM calcein AM and 5 mM CoCl_2_ for the final 15 min of the incubation. To detect the mitochondrial distribution, 50 nM MitoTracker CM-H_2_XRos were added during calcein loading. Calcein fluorescence was excited at 488 nm and emitted at 515 nm, and MitoTracker Red CM-H_2_XRos was excited at 579 nm and emitted at 599 nm.

For live cell imaging, cells were grown in culture dishes with a glass bottom. For time-lapse studies, they were placed in a temperature- and CO_2_-controlled chamber on the heating stage of a Zeiss Axio Observer. D1 microscope equipped with an Axiocam MRm monochrome digital camera was used to take photographs every 5 min for an observation period of 5 h (Carl Zeiss MicroImaging GmbH, Göttingen, Germany). Fluorescence intensity was analyzed with Axiovision LE software (Release 4.8 version).

### Transmission electron microscopy (TEM)

For TEM, collected cells were fixed in 2.5% glutaraldehyde with 0.1 M cacodylate buffer (pH 7.2) for 2 h at 4°C, washed twice with cold PBS, post-fixed in OsO_4_, dehydrated in graded ethanol, and embedded in Epon mixture. Sections were prepared with an ultra-microtome (MT-7000), mounted on copper grids, and counterstained with uranyl acetate and lead citrate. Photographs were taken using an electron microscope (HITACHI H-7600).

### Immunohistochemistry (IHC)

IHC was performed on 4-µm sections of formalin-fixed, paraffin-embedded tissues. Sections were deparaffinized in xylene and graded alcohol. Antigen retrieval was performed by autoclaving for 15 min. After incubation with blocking solution for 30 min, sections were incubated with an anti-Snail antibody [24], [25] for 1 h, a biotinylated secondary antibody for 20 min and then with streptavidin horseradish peroxidase (HRP) for 10 min. The antibody was visualized with diaminobenzidine (DAB) chromogen, and sections were counterstained with hematoxylin.

### Multicellular tumor spheroid (MTS) culture and staining

MCF-7, MDA-MB-231, and MDA-MB-361 cells were seeded at a density of 400 cells in 200 µl medium into 1.2% agarose-precoated 96-well plates. After 3 days of culture, 100 µl of medium was replaced with fresh medium every 2 days. To determine the MTS growth, diameters of spheroids were measured every day. MTSs were harvested and fixed in formalin. After processing into wax blocks, the spheroids were sectioned, and stained with hematoxylin and eosin, stained with HO/PI, or incubated with an anti-Snail antibody as described above.

### Spheroid selective dissociation

MTSs were dissociated into subpopulations of cells from different locations in the spheroid as described by LaRue *et al.* [37]. The spheroids were placed in a cylindrical chamber with a 70 um nylon mesh. After washing the spheroids with PBS, they were treated with a dissociation solution containing 0.125% trypsin (Life Technologies, Inc.) in a phosphate buffer containing 1 mM EDTA and 25 mM HEPES (pH 7.4). Cells dissociated from the spheroids were collected into stirred tubes containing complete medium on ice, while the remaining aggregates remained in the chamber. Cell suspensions were stored on ice until dissociation was complete. The cells isolated from different locations within spheroids were sonicated and then analyzed by Western blotting using antibodies against Snail and α-tubulin.

### Statistical analysis

All experiments were independently performed at least three times. Data were analyzed by the Student's *t*-test and *P*<0.05 was considered statistically significant.

## Results

### Snail is induced during metabolic stress-induced necrosis

**Figure 1:**
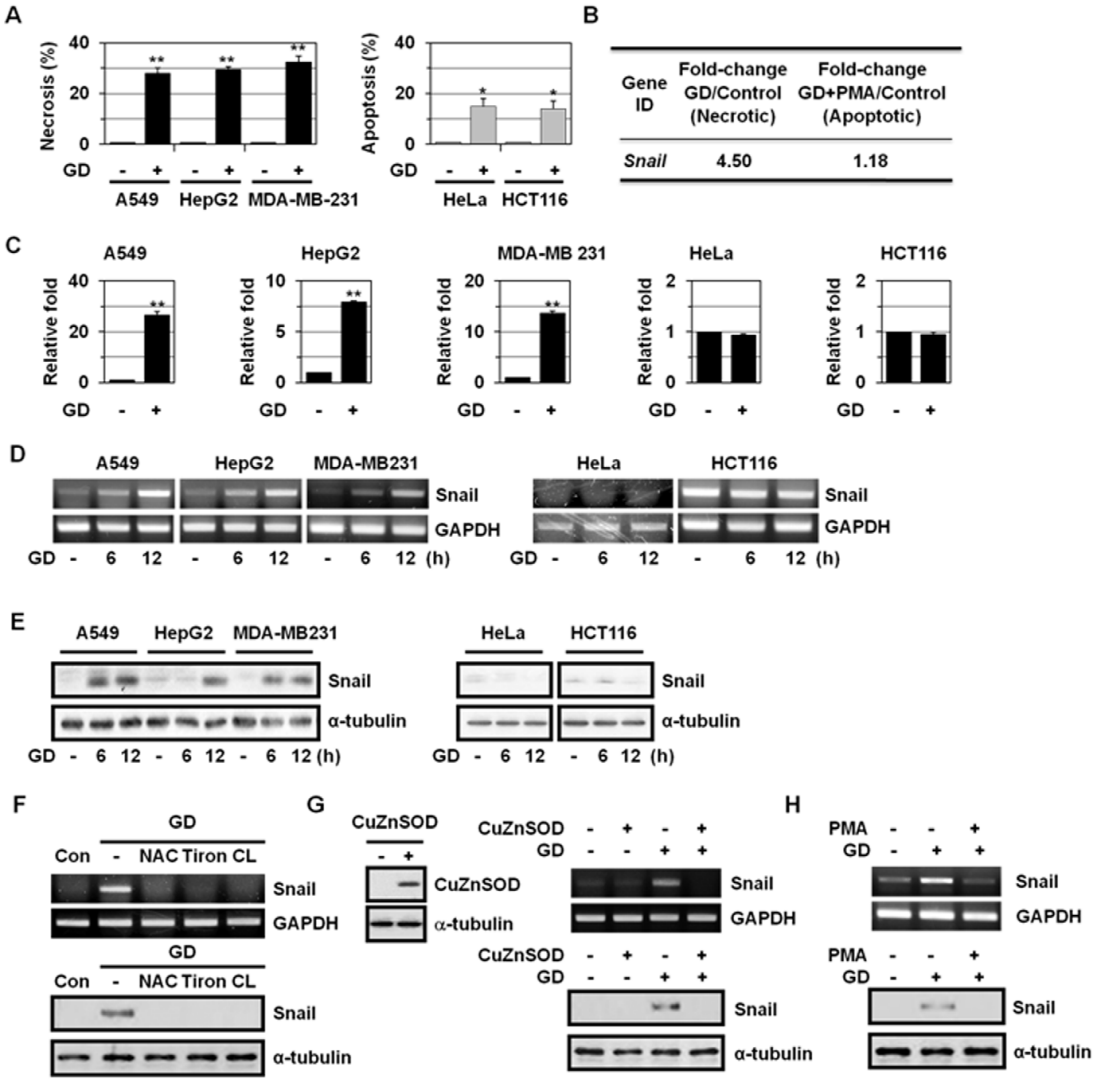
Induction of Snail during metabolic stress-induced necrosis. (A) A549, HepG2, MDA-MB-231, HeLa, and HCT116 cells were exposed to GD medium for 12 h, and the cells were stained with HO/PI and observed under a fluorescence microscope. Results are expressed as mean ± SE from 500 to 800 cells per treatment group and from three independent experiments. *P<0.05; **P<0.01 versus control. (B) A549 cells were pretreated with PMA (100 nM) for 30 min and washed with glucose-free RPMI 1640 twice and incubated in GD medium for 12 h. Microarray analysis was performed using fluorescently labeled cDNA probes prepared from total RNA. The numbers mean fold increase in expression over GD-untreated control cells. Data analysis was carried out using GeneSpring GX 7.3 (Agilent technologies). The values were normalized using the LOWESS algorithm. (C-E) A549, HepG2, MDA-MB-231, HeLa and HCT116 cells were cultured in normal growth medium or GD medium for the indicated times and then analyzed by real-time PCR for Snail and GAPDH. Values are normalized to GAPDH. Results are expressed as mean ± SE from three independent experiments. *P<0.05; **P<0.01 versus control (C). The cells were also analyzed by RT-PCR for Snail and GAPDH (D). The cells were also analyzed by Western blotting with antibodies against Snail and α-tubulin (E). (F) A549 cells were pretreated with antioxidants including NAC (10 mM), tiron (10 mM), and catalase (CL, 1000 U/ml) for 1 h, treated with GD medium for 12 h, and then analyzed by RT-PCR for Snail and GAPDH and Western blotting with antibodies against Snail and α-tubulin. (G) CuZnSOD-overexpressing A549 cells were treated with GD medium for 12 h and Snail expression was analyzed by RT-PCR for Snail and GAPDH and Western blotting with antibodies against Snail and α-tubulin. (H) A549 cells were pre-treated with 100 nM PMA for 30 min, exposed to GD medium for 18 h, and then analyzed by RT-PCR for Snail and GAPDH and Western blotting with antibodies against Snail and α-tubulin.

As demonstrated previously [35], GD induces cell death in cancer cells. The GD-induced cell death mode was determined by Hoechst 33342 (HO)/propidium iodide (PI) double staining. The DNA-binding dye HO crosses the plasma membrane of all cells that are viable or damaged, resulting in blue fluorescence within their nuclei, whereas PI only penetrates cells with damaged membranes, leading to nuclear red fluorescence. Thus, intact blue nuclei, condensed/fragmented blue nuclei, condensed/fragmented pink nuclei, and intact pink nuclei were considered viable, early apoptotic, late apoptotic (secondary necrotic), and necrotic cells, respectively. When treated with GD, the percentage of cells that had intact pink nuclei was significantly increased in A549 (28.0±2.1%), in HepG2 (29.5±1.1%), and in MDA-MB-231 (32.5±2.2%; Figure 1A and S1), indicating that GD induced necrosis. GD also induced release of HMGB1 and LDH into the extracellular space, confirming GD-induced necrosis in these cell lines. In contrast, GD induced apoptosis in HeLa (15.0±3.0%) and in HCT116 (14.0±3.0%; Figure 1A), as revealed by the presence of condensed/fragmented blue nuclei (Figure S1). Thus, in two-dimensional cultures, GD can induce either apoptosis or necrosis depending on the cell types due to their different cellular context.

Previously, we showed that in A549 cells, phorbol-12-myristate-13-acetate (PMA), a PKC activator, prevented GD-induced necrosis and switched the cell death mode to apoptosis by inhibiting ROS production, possibly by inducing manganese superoxide dismutase (MnSOD) expression and preventing GD-induced downregulation of copper-zinc superoxide dismutase (CuZnSOD) [35]. To identify the molecules that are involved in metabolic stress-induced necrosis, we analyzed the gene expression profiling of A549 cells that underwent necrosis or apoptosis under these conditions by cDNA microarrays. Of ∼3,096 genes analyzed, approximately 200 were up-regulated >2-fold and approximately 150 were down-regulated >2-fold (GEO accession no. GSE24271), indicating that gene expression pattern is changed during necrotic cell death. One of the GD-inducible genes was a zinc finger transcription factor, Snail (Figure 1B), which belongs to the Snail/Slug family of transcription factors [17]–[19]; Snail level was increased 4.5-fold during necrosis, whereas its level was not changed during apoptosis. Thus, Snail expression appeared to increase only in the presence of necrosis. Because Snail is known to promote tumor growth and progression and is induced during necrosis that plays a critical role(s) in tumor progression, we investigated the role of Snail in metabolic stress-induced necrosis.

Real-time quantitative PCR confirmed the induction of Snail by GD in A549 (26.5-fold), HepG2 (7.9-fold), and MDA-MB-231 cells (13.7-fold), but not in HeLa and HCT116 cells that underwent apoptosis upon GD (Figure 1C). RT-PCR and Western blot analysis also showed GD induction of Snail in A549, HepG2, and MDA-MB-231 cells, but not in HeLa and HCT116 cells (Figure 1D–E). Note that although HCT116 cells have high levels of Snail mRNA, they have low levels of Snail protein. Snail is a highly unstable protein with a half-life of only 25 min [23]. Thus, although Snail is highly expressed, it may be rapidly degraded in HCT116 cells. We observed that Snail induction by GD was inhibited by treatment with antioxidants, including N-acetytl cysteine (NAC), tiron, and catalase (Figure 1F); CuZnSOD overexpression (Figure 1G); or treatment with PMA (Figure 1H), which was previously shown to reduce ROS production in A549 cells [35]. These results indicate the redox-sensitivity of Snail expression.

### Snail is induced in the inner region of multicellular tumor spheroids

**Figure 2:**
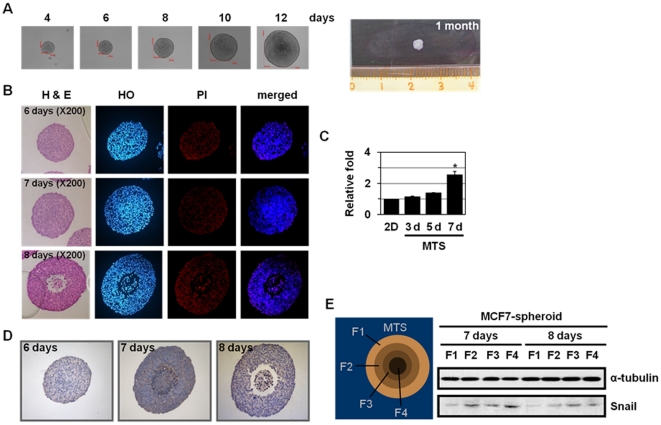
Induction of Snail in the inner region of multicellular tumor spheroids (MTSs). (A) Formation, growth and morphology of MTSs derived from MCF-7 cells. MCF-7 cells were seeded onto 1.2% agarose-coated 96-well plates at a density of 400 cells per well and cultured for up to 1 month. (B) MCF-7 cells were grown as MTSs, and the spheroids were sectioned and stained with H&E and HO/PI staining after 6–8 days of culture. (C) MCF-7 spheroids cultured on agarose for the indicated times were analyzed by real-time PCR using primers for Snail and GAPDH. Values are normalized to GAPDH. Results are expressed as mean ± SE from three independent experiments. *P<0.05 versus two-dimensional (2D)-cultured cells. (D) MCF-7 MTSs were sectioned and stained with an anti-Snail antibody after 6–8 days of culture. (E) After 7 days or 8 days of MCF-7 MTS culture, MTSs were dissociated into subpopulations of cells from different locations in the spheroids, as described in Materials and Methods. The cells isolated from different locations within MCF-7 spheroids were analyzed by Western blotting using antibodies against Snail and α-tubulin.

We examined Snail expression using MTSs, an *in vitro* model of solid tumors [38], [39]. MTSs closely mimic the growth characteristics of avascular regions of large solid tumors. With increasing size, MTSs develop a proliferation gradient, with proliferating cells at the periphery, cell-cycle arrested cells in inner regions, and necrotizing cells in core regions [38], [39]. The core regions lack oxygen and nutrients due to insufficient supply. These phenomena lead to necrotic cell death in the inner regions, forming the necrotic core [38], [39]. Three breast cancer cell lines, MCF-7, MDA-MB-231, and MDA-MB-361, were used to evaluate the formation of spheroids. As demonstrated previously [40], compared to MDA-MB-231 and MDA-MB-361cells, MCF-7 cells formed a tightly packed, rounded spheroids of a homogeneous size that required trypsin treatment and physical strength to disintegrate the spheroids (Figure 2A). These differences in compact MTS formation between three cell lines are likely due to their differential expression of surface adhesion molecules, such as E-cadherin or N-cadherin [39]. The size of MCF-7 MTSs reached approximately 3 mm after 1 month culture. To detect the necrotic core formation during MTS culture, MTSs were paraffin-sectioned and stained by H&E staining and HO/PI double-staining (Figure 2B). The PI-positive cells (with red nuclei) were analyzed in four discrete regions within the spheroid, with each region representing about 25% of the total spheroid size (as indicated in Figure 2E); the results of PI-positive cell count are summarized in Table S2. As shown in Jeong *et al.* [40], PI-positive cells were detected in 8 day MTSs but not in 6-7 day MTSs (Figure 2B and Table S2). In 8 day MTSs, PI-positive cells were observed in innermost F4 region (31 of 35 cells; 87.9%) and, to a lesser extent, in inner F3 region (6 of 155 cells; 3.7%), indicating that a necrotic core was formed beginning at 8 days in MCF-7 spheroids, when the spheroids reached approximately 700 µm in diameter.

Next, we examine the changes in Snail expression during MTS culture. Snail level was increased with extended MTS culture (Figure 2C); 2.6-fold Snail induction was observed at 7 day MTSs that may experience metabolic stress. We further examined the distribution of Snail in MTSs. For this purpose, the MTSs were stained by anti-Snail antibody in paraffin sections (Figure 2D). Snail-positive cells were analyzed in four discrete regions within the spheroid; the results of Snail-positive cell count are summarized in Table S3. As shown in Table S3, Snail-positive cells were detected in 8 day MTSs; prominent Snail staining was observed in the innermost F4 region (41 of 43 cells; 94.4%) and, to a lesser extent, in the inner F3 region (11 of 99 cells; 12.6%). To confirm the expression of Snail in MTSs, the spheroids were selectively dissociated to yield cells from four discrete regions within the spheroid. Snail was detected in the F2, F3, and the innermost F4 fractions, and was barely detectable in the outermost F1 fraction (Figure 2E). These results support that Snail expression is closely linked to metabolic stress, such as hypoxia and GD.

### Snail expression in hypoxic and glucose-depleted areas in solid tumors

**Figure 3:**
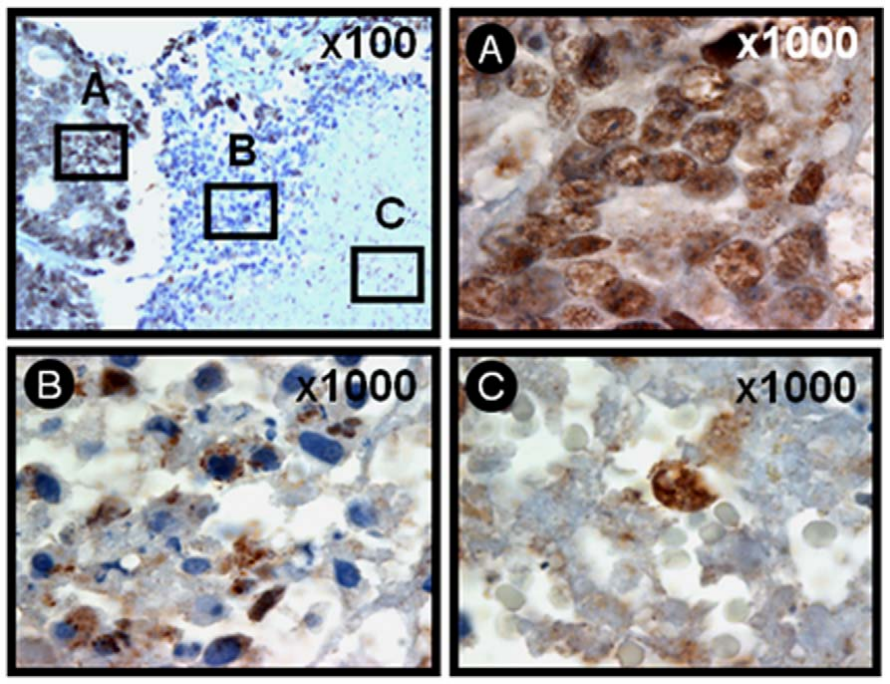
Immunohistochemical detection of Snail in metastatic colonic carcinoma in the liver. IHC was performed on 4-µm sections of formalin-fixed, paraffin-embedded tissues. Sections were deparaffinized as described in ‘Materials and Methods’ and were incubated with an anti-Snail antibody and the antibody was visualized with diaminobenzidine (DAB) chromogen, and sections were counterstained with hematoxylin. Snail, brown staining; nuclei, blue staining. A, the viable region of the tumor; B and C, the necrotic core region of the tumor.

We examined the patterns of Snail protein in human tumors, including metastatic colonic carcinoma in liver, colonic adenocarcinoma, and pulmonary adenocarcinoma, by IHC (Figure 3). The results of Snail-positive cell count are summarized in Table S4. We observed Snail expression in the inner region (in both para-necrotic region (region A) and the necrotic core (regions B and C)) of metastatic colonic carcinoma in liver. In para-necrotic regions, almost all tumour cells were viable and Snail-positive; Snail was detected in nuclei. In contrast, in necrotic regions, Snail was detected either in nuclei (13 of 235 cells; 5.5% of the necrotizing cells) or in cytosol as an aggregated form (21 of 235 cells; 8.9% of the necrotizing cells) or a diffused form (198 of 235 cells; 84.3% of the necrotizing cells; Table S4). Note that Snail aggregates in the necrotic regions were detected as either small dots or amorphous structures, which were different in size and were randomly dispersed or aggregated to each other in the cytosol. Similar results were obtained with colonic adenocarcinoma and pulmonary adenocarcinoma (data not shown). As normal epithelial cells grow and form solid tumor *in situ*, the cells in the inner regions experience hypoxic and GD stresses. Thus, these results further support that Snail is induced by metabolic stress.

### Snail shRNA prevents metabolic stress-induced necrosis in two dimensional cell culture

**Figure 4:**
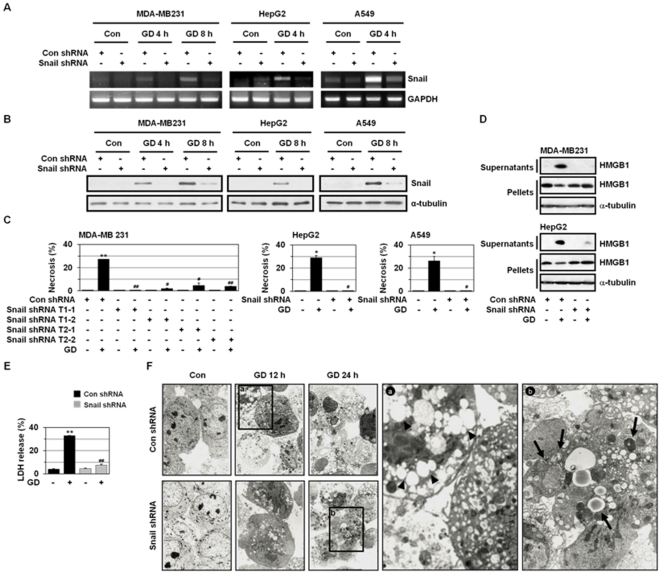
Snail shRNA prevents metabolic stress-induced necrosis. (A, B) MDA-MB-231, HepG2, and A549 cells that were stably transfected with control or Snail shRNA were cultured in normal growth medium or GD medium for the indicated times and then analyzed by RT-PCR using primers for Snail and GAPDH (A). The cells were also analyzed by Western blotting with antibodies against Snail and α-tubulin (B). (C) MDA-MB-231, HepG2, and A549 cells that were stably transfected with control or Snail shRNA were cultured in normal growth medium or GD medium for 12 h, stained with HO/PI, and observed with fluorescence microscopy, and apoptotic and necrotic cells were scored. Results are expressed as mean ± SE from 500 to 800 cells per treatment group and from three independent experiments. *P<0.05; **P<0.01 versus control, ^#^ *P*<0.05; ^##^ *P*<0.01 versus control shRNA. To ascertain the activity of Snail shRNA, we used two independent Snail-specific shRNAs (targets 1 (T1) and 2 (T2) as described in Materials and Methods; T1-1 and T1-2 indicate two different clones produced after G418 selection process). These two independent Snail shRNAs showed similar effects on GD-induced necrosis, etc. The data obtained using target 1 Snail shRNA are shown throughout all of the figures. (D) MDA-MB-231 and HepG2 cells that were stably transfected with control or Snail shRNA were treated with GD medium for 12 h and both the medium bathing the cells (supernatants) and the cells (pellets) were prepared and analyzed by Western blotting with antibodies against HMGB1 and α-tubulin. (E) MDA-MB-231 cells that were stably transfected with control or Snail shRNA were treated with GD medium for 12 h and analyzed by lactate dehydrogenase (LDH) release. Results are expressed as mean ± SE from three independent experiments. **P<0.01 versus control, ^##^ *P*<0.01 versus control shRNA. (F) TEM for HepG2 cells stably transfected with control or Snail shRNA that were treated with GD medium for the indicated times. The large TEM image in the right panel is the enlargement of the box in the left panel (panel a, control shRNA/GD 12 h; panel b, Snail shRNA/GD 24 h). Arrowheads in panel **a** indicate cytoplasmic clear vacuolar structures. Arrows in panel **b** indicate autophagic vacuole-like structures.

We investigated whether Snail is functionally linked to GD-induced necrosis in A549, HepG2, and MDA-MB-231 cell lines by specific transcript knockdown with short hairpin RNA (shRNA). We employed two different shRNA oligonucleotides; one (target 1) is a 19-mer shRNA oligonucleotide directed to the N-terminal region of the first zing finger of human Snail mRNA sequences (position from 129 to 147 in human cDNA, accession number NM005985) [28] and another (target 2) is a 21-mer shRNA oligonucleotide directed to the N-terminal region of human Snail mRNA sequences (position from 12 to 32 in human cDNA, accession number NM005985). These two oligonucleotides were not directed to human Slug/Snail2 mRNA. Snail shRNA was verified to be effective in knocking down Snail mRNA levels, whereas it did not cause a significant change in the level of endogenous Slug mRNA (Figure S2), proving that Snail shRNA is specific to Snail and is useful to abrogate its function. In addition, Snail shRNA also prevented GD induction of Snail, as determined by RT-PCR and Western blotting (Figure 4A–B).

Transcript knock-down by stable Snail shRNA significantly blocked GD-induced necrosis in MDA-MB-231, HepG2, and A549 cells, as revealed by a prominent decrease in the population of cells that had intact pink nuclei in HO/PI staining (Figure 4C and S3). In addition, Snail shRNA suppressed GD-induced release of HMGB1 into the extracellular space (Figure 4D). Snail shRNA transfection also prevented necrosis-linked LDH release (Figure 4E). Ultrastructurally, Snail shRNA prevented GD-induced membrane rupture and necrotic cytoplasmic clear vacuoles without the contents undergoing digestion, which has been described in necrotic cell death (Figure 4F). When GD-induced necrosis was inhibited by Snail knockdown, apoptosis did not occur as an alternative death mechanism. Instead, autophagic vacuole-like structures with contents undergoing digestion were observed (Figure 4F). These results suggest that Snail may play a critical role(s) in metabolic stress-induced necrosis.

Snail induces EMT in cancer cells [17]–[19] but does not trigger necrosis (data not shown), indicating that Snail is necessary but not sufficient for metabolic stress-induced necrosis. Because necrosis is accompanied by several different processes, including mitochondrial dysfunction, excess ROS production, and ATP depletion [12], [16], Snail may trigger necrosis if tumor cells are under such metabolic stress.

### Snail shRNA inhibits necrosis in multicellular tumor spheroid

**Figure 5:**
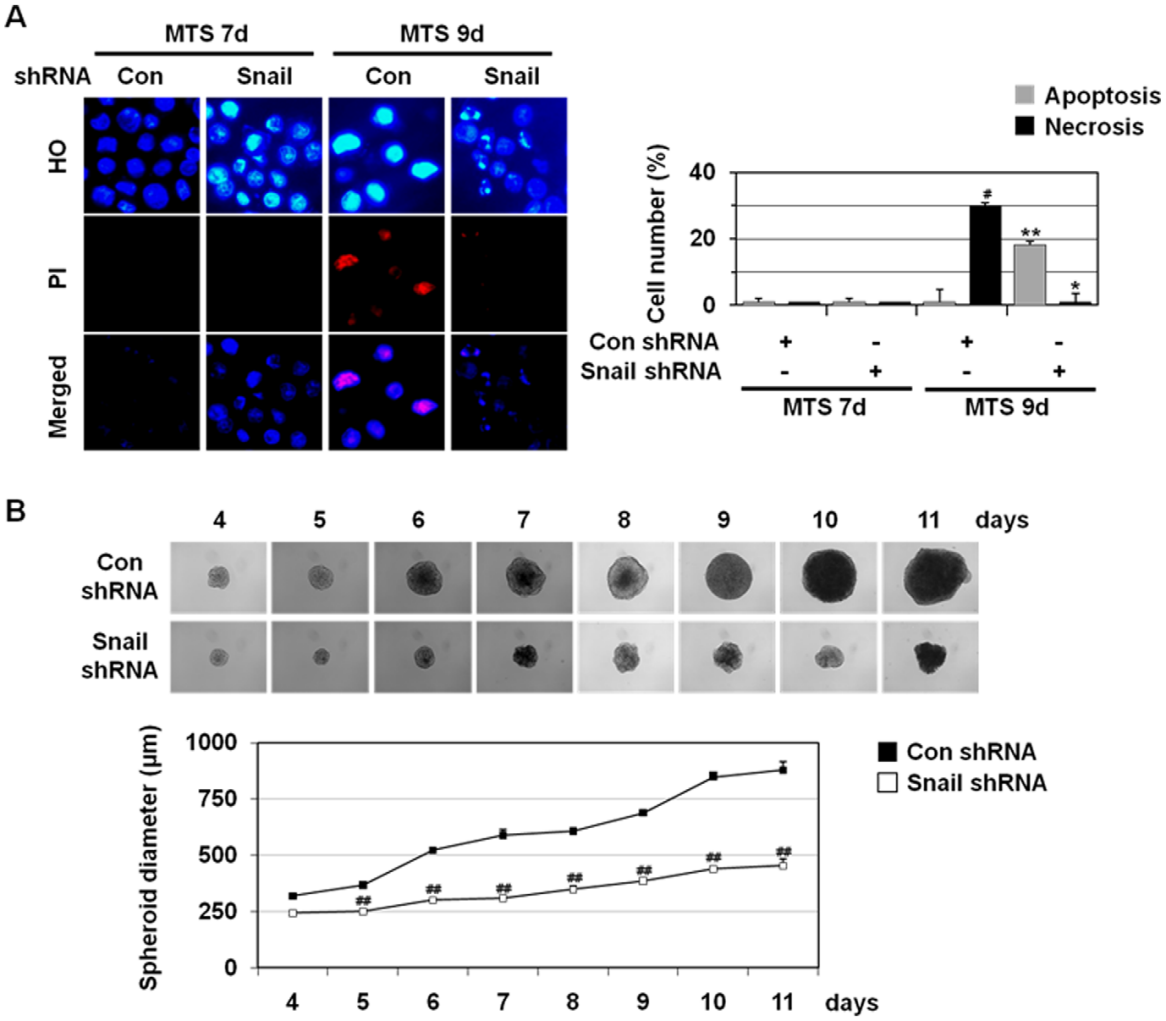
Snail shRNA prevents necrosis in MTS. (A) MCF-7 cells stably transfected with control and Snail shRNA were seeded into 1.2% agarose-coated 96-well plates at a density of 400 cells per well and cultured for 7 and 9 days. Then the cells were dissociated and stained with HO/PI and observed with fluorescence microscopy (left) and apoptotic and necrotic cells were scored (right). Results are expressed as mean ± SE from 500 to 800 cells per treatment group and from three independent experiments. ^#^ *P*<0.05; ^##^ *P*<0.01 versus control shRNA. (B) Formation, growth and morphology of MTSs from MCF-7 control and Snail shRNA stable cells were measured. To calculate MTS size, diameters of five spheroids were measured every day. Results are expressed as mean ± SE from three independent experiments. ^##^ *P*<0.01 versus control shRNA.

Continued spheroid growth leads to the formation of necrotic core due to microenvironmental stresses including deprivation of oxygen and nutrients. HO/PI staining using total cells obtained from MTSs revealed the occurrence of necrosis (29.8±3.6%) at 9 day culture of control shRNA MCF-7 MTSs (Figure 5A). We examined the effects of Snail shRNA on the necrotic cell death in MTSs. Snail shRNA appeared to suppress the occurrence of necrotic cell death. A prominent anti-necrotic activity of Snail shRNA was observed at 9 day in MCF-7 MTS culture. Snail shRNA prevented necrosis (0%) and switched the cell death mode to apoptosis (18.2±2.4%), as revealed by a prominent increase in the population of cells that had condensed/fragmented blue nuclei in HO/PI staining (Figure 5A).

We also observed that stable Snail silencing in MCF-7 MTSs suppressed the growth of the MCF-7 MTSs (Figure 5B), supporting a critical role(s) of Snail in tumor growth. Similar results were reported in solid tumors [33]; silencing Snail by stable RNA interference in carcinoma cell lines leads to a dramatic reduction of *in vivo* tumor growth.

### Snail shRNA prevents metabolic stress-induced mitochondrial ROS production, loss of mitochondrial membrane potential (ΔΨm), and mitochondrial permeability transition (mPT)

**Figure 6:**
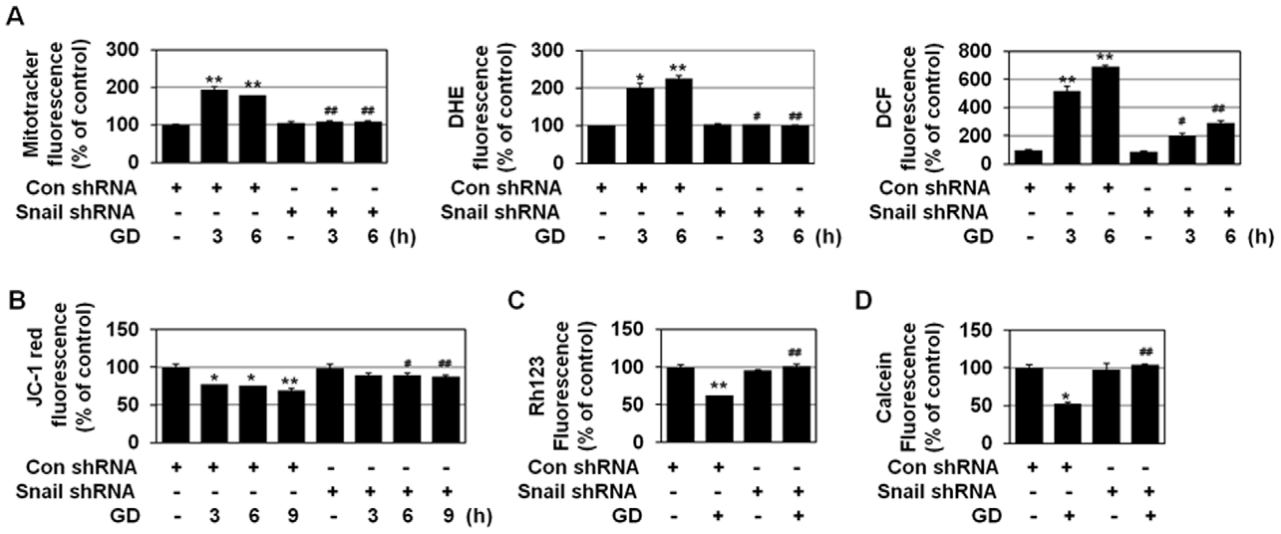
Snail shRNA prevents metabolic stress-induced ROS production, loss of ΔΨm, and mPT. (A) MDA-MB-231 cells that were stably transfected with control or Snail shRNA were exposed to GD medium for 3 h or 6 h, and mitochondrial ROS and O_2_^-^ and intracellular H_2_O_2_ production was measured using the MitoTracker Red CM-H_2_XRos, HE, and DCFH-DA, respectively, under a confocal microscope (X200, Carl Zeiss, LSM510). Results are expressed as mean ± SE from approximately 200 cells per treatment group and from three independent experiments. *P<0.05; **P<0.01 versus control, ^#^ *P*<0.05; ^##^ *P*<0.01 versus control shRNA. (B) MDA-MB-231 cells stably transfected with control or Snail shRNA were incubated with GD for the indicated times and then treated with 5 mg/ml JC-1 for 15 min, and observed by fluorescence microscopy. Results are expressed as mean ± SE from 50 to 100 cells per treatment group and from three independent experiments. *P<0.05; **P<0.01 versus control, ^#^ *P*<0.05; ^##^ *P*<0.01 versus control shRNA. (C) MDA-MB231 cells that were stably transfected with control or Snail shRNA were incubated with GD for 9 h; loaded with 5 µM rhodamine 123; and observed under a fluorescence microscope. Results are expressed as mean ± SE from 50 to 80 cells per treatment group and from three independent experiments. **P<0.01 versus control, ^##^ *P*<0.01 versus control shRNA. (D) MDA-MB231 cells that were transiently transfected with control or Snail shRNA were incubated in normal medium or GD medium for 9 h and loaded with 0.5 µM calcein AM and 5 mM CoCl_2_ for the final 15 min of the incubation. To detect cytoplasmic mitochondrial distribution, 50 nM MitoTracker CMX-ROS were added during calcein loading. Calcein fluorescence was excited at 488 nm and emitted at 515 nm, MitoTracker Red CMX-ROS was excited at 579 nm, and emitted at 599 nm, and the cells were observed by fluorescence microscopy. Results are expressed as mean ± SE from 15 to 30 cells per treatment group and from three independent experiments. *P<0.05 versus control, ^##^ *P*<0.01 versus control shRNA.

Mitochondrial ROS plays a crucial role(s) in GD-induced cytotoxicity [41], [42] and necrosis in human cancer cells [35]. GD significantly enhanced the production of mitochondrial ROS, O_2_^-^, and intracellular H_2_O_2_ (Figure 6A and S4), as revealed by staining with three different fluorogenic probes: MitoTracker Red CM-H_2_XRos, HE, and DCFH-DA. Measurement of fluorescence intensity revealed that mitochondrial ROS, O_2_^-^, and intracellular H_2_O_2_ levels were increased upon GD and Snail interference blocked GD-induced production of these intracellular ROS (Figures 6A, S4 and S5), indicating that Snail may control necrosis by regulating metabolic stress-induced ROS production.

We also monitored the ΔΨm of cells with JC-1, a mitochondria-specific and lipophilic cationic fluorescence dye. Upon GD treatment, red J-aggregate fluorescence was progressively lost, and cytoplasmic diffusion of green monomer fluorescence was detected; Snail shRNA inhibited the effects of this GD-induced decline in ΔΨm (Figure 6B and S6). To detect changes in ΔΨm, cells were also stained with rhodamine 123. In response to GD treatment, cells quickly lost the punctate fluorescence pattern of rhodamine 123, and Snail shRNA prevented this ΔΨm disruption (Figure 6C).

Cobalt-quenched calcein (CoQC) measurement was used to determine mPT. Upon GD treatment, calcein fluorescence was lost following the opening of the mPT pore, and Snail shRNA prevented this mPT pore opening (Figure 6D and S6).

Our results show that Snail may regulate mitochondrial ROS production, loss of ΔΨm, and mPT upon metabolic stress, leading to potentially significant mitochondrial and cellular injury.

Mitochondrial O_2_^-^ is produced mostly at Complex I or Complex III of the electron transport chain even under normal conditions [43], [44], its production is enhanced by GD treatment and triggers necrotic cell death. Cellular redox status is regulated by the levels of antioxidant enzymes, such as SOD and catalase, as well as the efficacy of the ROS-generating system, specifically the mitochondria. Thus, we examined the effects of Snail on antioxidant expression. However, Snail did not affect cellular levels of CuZnSOD, MnSOD or catalase (Figure S7A). In addition, Snail shRNA did not prevent menadione (an O_2_^-^ generator)-induced necrosis (Figure S7B), indicating that Snail may not affect antioxidant levels.

## Discussion

### ROS-dependent induction of Snail

The transcriptional factor Snail is induced in response to several kinds of tumor-promoting cytokines and growth factors, such as TGFβ and Wnt in many human carcinomas and regulates epithelial-mesenchymal transition, which has essential role(s) in tumor invasion and progression [17]–[19]. Indeed, Snail has been detected at the invasive front of epidermoid carcinomas, and has been associated with invasiveness of ductal breast carcinomas and hepatocarcinomas [17]–[19], [25]–[27]. In this study, we showed that it could be induced in response to metabolic stress in the culture of cancer cells, including A549, HepG2, and MDA-MB-231 (Figure 1). Snail expression was also detected in the inner regions (F3 and F4) of MTS system, an *in vitro* model of solid tumors (Figure 2), and in the inner regions of human tumors, which experience metabolic stress (Figure 3), supporting that Snail is induced by metabolic stress. Metabolic stress-induced Snail expression appeared to occur in a ROS-dependent manner (Figure 1). Similar ROS-dependent Snail induction has been reported in hepatocellular carcinoma cells [45] and in MMP-3/Rac1b signal cascade-mediated EMT of mouse mammary epithelial cells [46]. We also observed that Snail expression is increased by H_2_O_2_ (300 µM) or menadione (10 µM, an O_2_^-^ generator) (data not shown). PI3K/Akt-mediated inhibition of GSK3β has been demonstrated to be responsible for Snail expression by ROS such as H_2_O_2_ [45].

In addition to its expression in the inner regions (F3 and F4) of MTS system, Snail expression was also detected in the F2 region of 7 and 8 day MTSs (Figure 2). Because increased levels of p27Kip1, a CKI family protein that is regulated by HIF-1α and is involved in the regulation of G1-S progression, is detected in the F2 and F3 regions [37], the F2 and F3 regions are likely to represent a hypoxic region. These results suggest that Snail could be induced by hypoxia. In fact, hypoxia is known to induce mRNA expression of Snail in ovarian cancer cell lines [47]. Snail expression by hypoxia has been suggested to be mediated by hypoxia-inducible factor 1 (HIF1) itself as well as HIF1-upregulated TGF-Smad-mediated signal cascade [48]. Moreover, hypoxia increases the levels of Snail protein through HIF-1/Twist-dependent down-regulation expression of FBXl14, which promotes Snail ubiquitinylation and proteasome degradation, showing that Snail protein stability could be dynamically regulated by a GSK-3β/β-TrCP1-independent process [49]. In addition, hypoxia also induces Snail translocation into the nucleus through ROS-dependent inhibition of GSK-3β [50], [51]. These results support that Snail expression is related to microenvironmental stresses, such as hypoxia and GD.

### Implication of Snail in metabolic stress-induced necrosis

We showed that Snail shRNA inhibited metabolic stress-induced increase in PI-positive cell population, HMGB1 release, and LDH release in two-dimensional cell culture (Figure 4), indicating that Snail is implicated in metabolic stress-induced necrosis. The anti-necrotic effects of Snail shRNA on necrotic cell death were also observed in MTSs, with its prominent anti-necrotic activity at 9 day in MCF-7 MTS culture (Figure 5). In two-dimensional cell culture, while inhibiting necrosis, Snail shRNA switched GD-induced cell death mode to autophagy-like cell death but not apoptosis (Figure 4). Under this circumstance, apoptosis is not likely to occur because Snail shRNA prevented ROS production that is also necessary for induction of apoptosis. In case of MCF-7 MTSs, while preventing necrosis, Snail shRNA switched the cell death mode to apoptosis (Figure 5). The stress conditions are quite different in two-dimensional and MTS cultures; GD in two-dimensional culture and OGD in MTSs. Thus, shRNA appeared to switch necrotic cell death to either autophagy-like cell death or apoptosis depending on stress conditions in two-dimensional and MTS cultures.

Snail knock-down also inhibited MTS growth (Figure 5). Snail has been shown to block the cell cycle [29] and Snail overexpressing cells had significantly longer population doubling times compared with the vector control cells (33.8 h and 27.7 h, respectively, data not shown). Thus, Snail shRNA-mediated MTS growth inhibition cannot be explained by the effects of Snail on the cell cycle and may occur via unknown mechanism of Snail shRNA other than its effects on the cell cycle. Even though Snail shRNA MTS had smaller size at 9 day (453±30 µm), the size is likely to be enough to experience metabolic stress in core regions (Figure 2A, B), because 9 day Snail shRNA MTS underwent cell death (apoptosis instead of necrosis). In fact, a necrotic core is known to be formed in most spheroids larger than 400–500 µm [52].

### Snail shRNA-mediated necrosis inhibition is linked to its ability to suppress metabolic stress-induced mitochondrial ROS production

Mitochondrial O_2_^-^ is known to mediate GD-induced cytotoxicity and cell death [41], [42], [53]. ROS can induce either apoptosis or necrosis in human cancer cells, depending on the level of insult; low levels of ROS induce apoptosis, whereas higher levels induce necrosis. ROS could induce the mPT in the mitochondrial inner membrane; while the mPT pore opening could induce apoptosis by causing the release of mitochondrial apoptotic molecules, its prolonged opening results in necrotic cell death [54]. Furthermore, mPT leads to the loss of mitochondrial membrane potential (ΔΨm). When the mPT pore is open for longer periods, cells cannot generate ATP by oxidative phosphorylation, leading to necrotic cell death as a consequence of ATP depletion. In addition, ROS could induce insoluble protein aggregates that are toxic to cells and cause cell death especially necrosis through triggering the necrosis-associated membrane rupture. In this study, we found that Snail exists as aggregates that are randomly dispersed in the cytosol in necrotizing cells of metastatic colonic carcinoma in liver (Figure 3). Previously, we have shown using different experimental approaches including subcellular fractionation, RIPA-solubility assay, immunofluorescence microscopy, and TEM that several cellular proteins including p53, caspase-3, caspase-9, beclin 1, and Snail are ROS-dependently aggregated in an insoluble form in the cytosol during GD-induced necrosis [55]. Thus, the immunopositive materials in Figure 3 are thought to be in the form of protein aggregates based on previous findings. Similar pattern of protein aggregates has been demonstrated in focal ischemic region in brain, which is similar to OGD region found in tumors [56]–[58]. Ring-like structures among protein aggregates (oligomeric globular assemblies, protofibrils, and ring-like structures) could form non-specific membrane pores that lead to necrosis [59]. Here, we showed that shSnail inhibits metabolic stress-induced ROS production, metabolic stress-induced loss of mitochondrial membrane potential and mitochondrial permeability transition (Figure 6). Furthermore, Snail shRNA was observed to block metabolic stress-induced protein aggregation [55]. Thus, Snail shRNA may exert its anti-necrotic effects through preventing metabolic stress-induced loss of mitochondrial membrane potential, mitochondrial permeability transition, and metabolic stress-induced protein aggregation, which are the primary events that trigger necrosis, through its inhibitory effect on mitochondrial ROS production.

How does Snail control mitochondrial ROS production in response to GD? Mitochondrial dysfunction has been linked to the induction of necrosis. Tumor cells have been shown to have abnormal mitochondrial structure and DNA integrity and high rates of mtDNA mutations [60], [61], and this has been suggested to make the cells more sensitive to oxidative stress and cell killing induced by GD or treatment with 2-deoxyglucose, which inhibits glycolysis [53]. In addition, tumor cells with dysregulated mitochondria undergo necrosis instead of apoptosis in response to alkylating DNA damage that induces rapid ATP depletion through PARP activation [62]. We observed that metabolic stressed-induced Snail protein aggregates are colocalized with mitochondria, possibly in inactive form in which its transcriptional activity was impaired [55]. Thus, we speculate that Snail aggregates may affect mitochondrial function and sensitize tumor cells to metabolic stress and death by necrosis. The molecular mechanism underlying regulation of mitochondrial function by Snail is under investigation.

Our results showed that the GD-induced expression of Snail is ROS-dependent (Figure 1), and the GD-induced production of ROS is also Snail-dependent (Figure 6). ROS produced under stress conditions are known to spread from one mitochondrion to neighboring mitochondria in a process known as ROS-induced ROS release (RIRR), constituting a positive feedback mechanism for enhanced ROS production leading to mitochondrial and cellular injury [63], [64]. The induction of mitochondrial ROS up to a critical threshold level has been suggested to be a key step for propagation of the synchronized RIRR response. GD-induced Snail (including an aggregated form) may cause mitochondrial dysfunction, facilitating ROS production in response to GD and the increased ROS may in turn enhance Snail expression to accelerate massive ROS production by RIRR and to induce GD-induced cytotoxicity and necrosis, thereby forming a positive feedback loop between Snail expression and cellular ROS levels. We observed that although Snail shRNA could only suppress Snail expression by ∼50% (Figure S2), it prevented almost completely GD-induced mitochondrial ROS production (Figure 6). In accordance with this observation, GD-induced Snail expression was also completely inhibited by Snail shRNA (Figure 4). We speculate that Snail may be slightly induced (in spite of the knock-down effect of shRNA on Snail expression) by GD treatment in Snail shRNA cells, but this small amount of Snail induction may less affect mitochondrial function with very low level of ROS production, which does not reach to a threshold level to trigger RIRR for massive ROS production and subsequent further induction of Snail by GD.

### Biological relevance of Snail regulation of necrosis

In solid tumors, cells that are not adapted to metabolic constraints such as hypoxia and GD die by necrosis as ‘a reparative cell death’ that could promote tumor progression by releasing the tumor-promoting cytokine HMGB1 [1]–[6]. Snail plays a critical role(s) in tumor progression by inducing EMT, tumor cell invasion and metastases via downregulating E-cadherin [17]–[19] and protecting cells from apoptosis induced by many pro-apoptotic stimuli [28]–[32]. Our results suggest that in the absence of metabolic stress, Snail triggers EMT for tumor invasion, but in the presence of metabolic stress, it may facilitate metabolic stress-induced necrosis by promoting mitochondrial ROS production. Thus, Snail may contribute to tumor progression by promoting necrosis in addition to inducing EMT and preventing apoptosis. Taken together, our findings demonstrate that Snail is implicated in metabolic stress-induced necrosis, providing a new function for Snail in tumor progression.

## Supporting Information

Figure S1 **Induction of Snail during metabolic stress-induced necrosis.** (A) A549, HepG2, MDA-MB-231, HeLa, and HCT116 cells were exposed to GD medium for 12 h, and the cells were stained with HO/PI and observed under a fluorescence microscope. Arrow indicates apoptotic cells, * indicates necrotizing cells.
(TIF)

Figure S2 **Snail interference is no effect in the level of endogenous Slug mRNA.** MDA-MB-231 cells that were stably transfected with control or Snail shRNA analyzed by real-time PCR for Snail, Slug and GAPDH. Values are normalized to GAPDH. Results are expressed as mean ± SE from three independent experiments. ^#^ *P*<0.05; ^##^ *P*<0.01 versus control shRNA.
(TIF)

Figure S3 **Snail shRNA prevents metabolic stress-induced necrosis.** MDA-MB-231, HepG2, and A549 cells that were stably transfected with control or Snail shRNA were cultured in normal growth medium or GD medium for 12 h, stained with HO/PI, and observed by fluorescence microscopy.
(TIF)

Figure S4 **Snail shRNA prevents metabolic stress-induced ROS production.** MDA-MB-231 cells that were stably transfected with control or Snail shRNA were exposed to GD medium for 3 h or 6 h, and mitochondrial ROS and O_2_^-^ and intracellular H_2_O_2_ production was measured using the MitoTracker Red CM-H_2_XRos, HE, and DCFH-DA, respectively, under a confocal microscope (X200, Carl Zeiss, LSM510).
(TIF)

Figure S5 **Snail shRNA prevents metabolic stress-induced mitochondrial ROS.** MDA-MB-231 control and Snail shRNA stable cells that were treated with 50 nm MitoTracker Red CM-H_2_XRos were exposed to GD medium and observed by time-lapse microscopy (X400, Carl Zeiss, Axio Observer.D1). Fluorescence images were taken every 5 min for 5 h, and fluorescence intensity was analyzed with Axiovision LE software (Release 4.8 version). Values represent average of total cellular MitoTracker Red CM-H_2_XRos fluorescence expressed as relative fluorescence intensity at 0 min.
(TIF)

Figure S6 **Snail shRNA prevents metabolic stress-induced loss of ΔΨm and mPT.** (A) MDA-MB-231 cells stably transfected with control or Snail shRNA were incubated with GD for the indicated times and then treated with 5 mg/ml JC-1 for 15 min, and observed by fluorescence microscopy. (B) MDA-MB231 cells that were transiently transfected with control or Snail shRNA were incubated in normal medium or GD medium for 9 h and loaded with 0.5 µM calcein AM and 5 mM CoCl_2_ for the final 15 min of the incubation. To detect cytoplasmic mitochondrial distribution, 50 nM MitoTracker CMX-ROS were added during calcein loading. Calcein fluorescence was excited at 488 nm and emitted at 515 nm, MitoTracker Red CMX-ROS was excited at 579 nm, and emitted at 599 nm, and the cells were observed by fluorescence microscopy.
(TIF)

Figure S7 **The effects of Snail on antioxidant expression and menadione-induced necrosis.** (A) The Snail tet-inducible cell line MCF-7 was treated with DOX for 48 h or 72 h and then analyzed by Western blotting with antibodies against Snail, CuZnSOD, MnSOD, catalase, and α-tubulin. (B) MDA-MB-231 cells that were stably transfected with control and Snail shRNA were cultured in normal growth medium in the presence or absence of 100 µM and 250 µM menadione, stained with HO/PI, and observed under a fluorescence microscope. Results are expressed as mean ± SE from 500 to 800 cells per treatment group and from three independent experiments.
(TIF)

Table S1 **Primer sequences for RT-PCR and real time PCR.**
(TIF)

Table S2 **Statistical analysis of HO-PI staining results (** **Figure 2B** **).**
(TIF)

Table S3 **Statistical analysis of immunohistochemistry results (** **Figure 2D** **).**
(TIF)

Table S4 **Statistical analysis of immunohistochemistry results in solid tumors (** **Figure 3** **).**
(TIF)
