# Supplementary figures and images for: Implication of Snail in Metabolic Stress-Induced Necrosis
Source: PLoS One. 2011 Mar 23;6(3):e18000. doi: 10.1371/journal.pone.0018000 (PMC3063248; doi:10.1371/journal.pone.0018000)

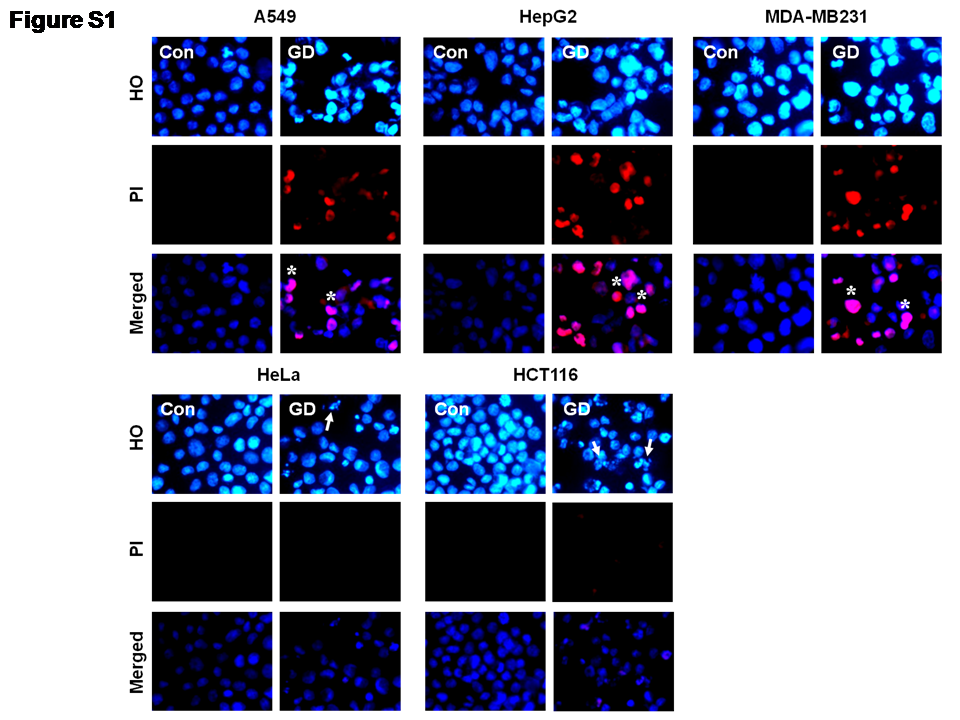

Supplement: Figure S1 — Induction of Snail during metabolic stress-induced necrosis. (A) A549, HepG2, MDA-MB-231, HeLa, and HCT116 cells were exposed to GD medium for 12 h, and the cells were stained with HO/PI and observed under a fluorescence microscope. Arrow indicates apoptotic cells, * indicates necrotizing cells. (TIF) [file pone.0018000.s001.tif]

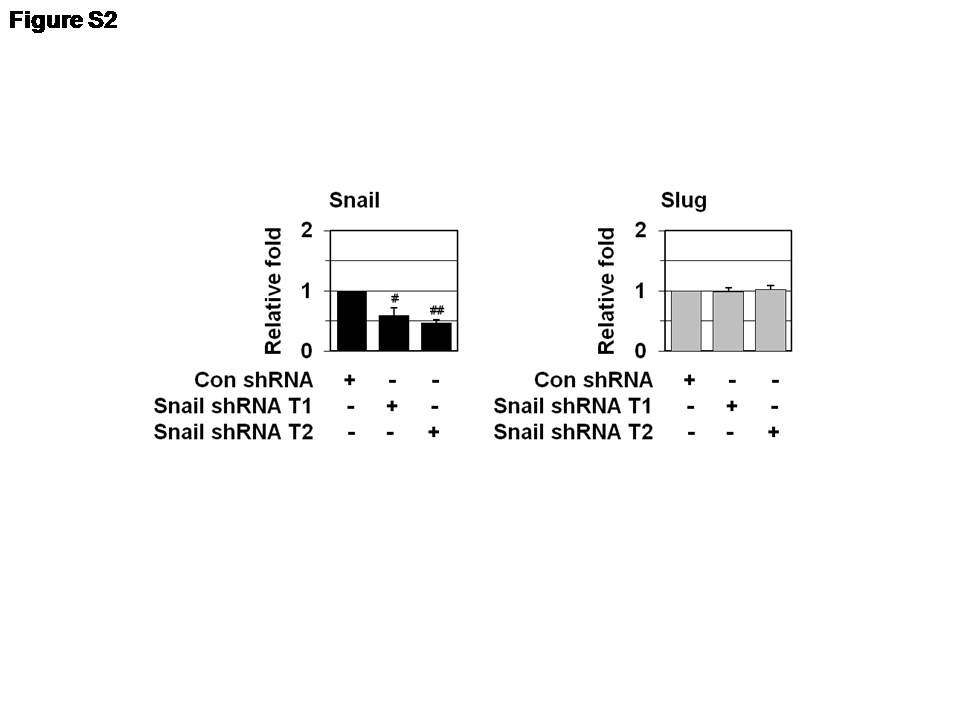

Supplement: Figure S2 — Snail interference is no effect in the level of endogenous Slug mRNA. MDA-MB-231 cells that were stably transfected with control or Snail shRNA analyzed by real-time PCR for Snail, Slug and GAPDH. Values are normalized to GAPDH. Results are expressed as mean ± SE from three independent experiments. # P<0.05; ## P<0.01 versus control shRNA. (TIF) [file pone.0018000.s002.tif]

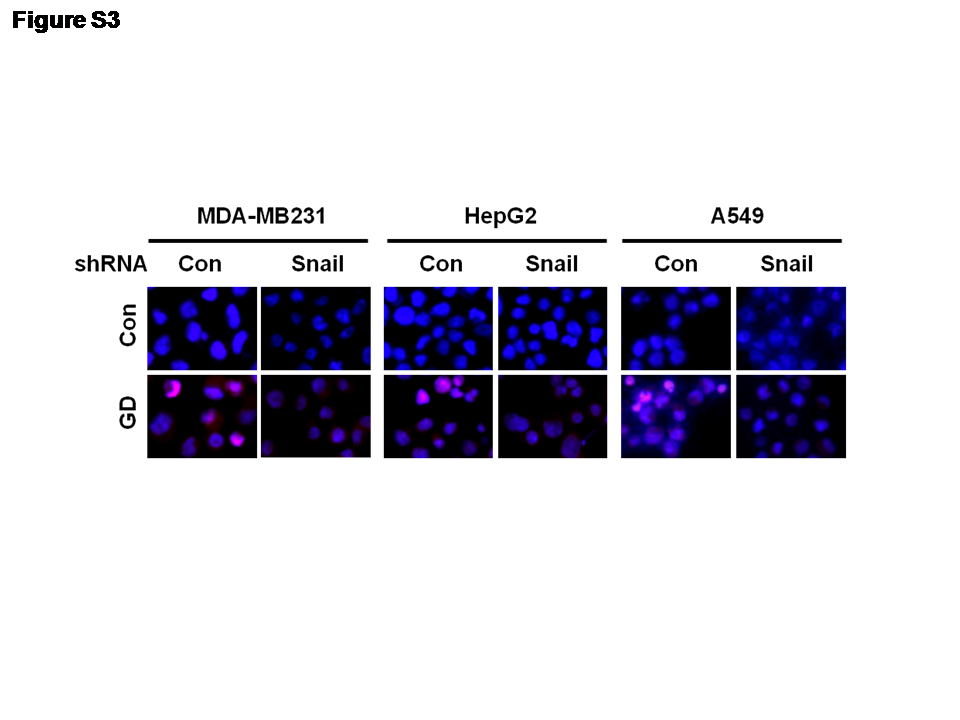

Supplement: Figure S3 — Snail shRNA prevents metabolic stress-induced necrosis. MDA-MB-231, HepG2, and A549 cells that were stably transfected with control or Snail shRNA were cultured in normal growth medium or GD medium for 12 h, stained with HO/PI, and observed by fluorescence microscopy. (TIF) [file pone.0018000.s003.tif]

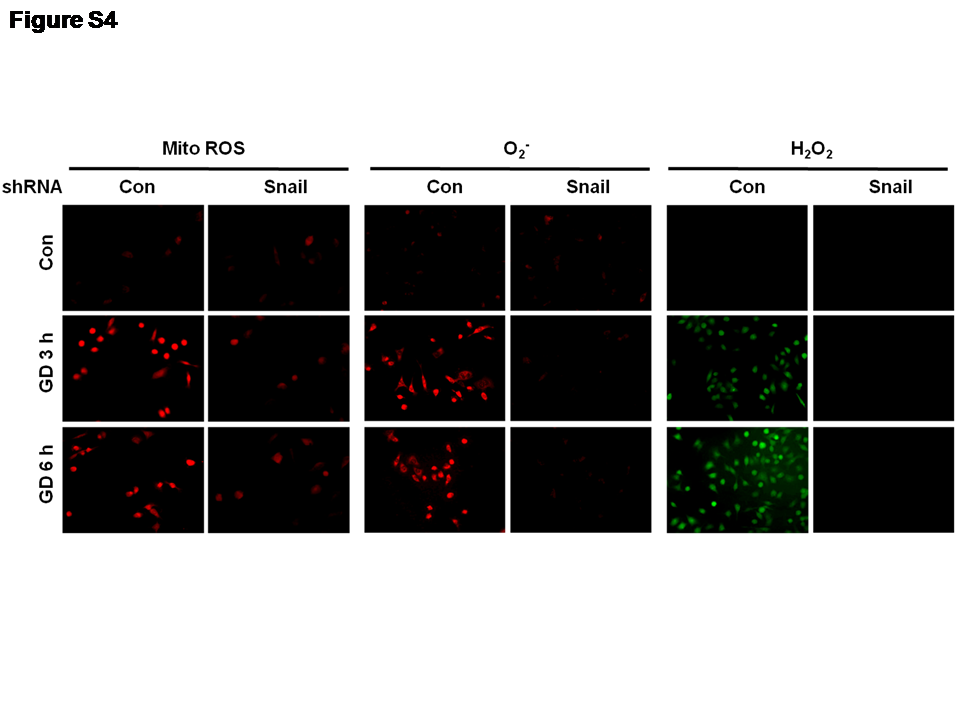

Supplement: Figure S4 — Snail shRNA prevents metabolic stress-induced ROS production. MDA-MB-231 cells that were stably transfected with control or Snail shRNA were exposed to GD medium for 3 h or 6 h, and mitochondrial ROS and O2 - and intracellular H2O2 production was measured using the MitoTracker Red CM-H2XRos, HE, and DCFH-DA, respectively, under a confocal microscope (X200, Carl Zeiss, LSM510). (TIF) [file pone.0018000.s004.tif]

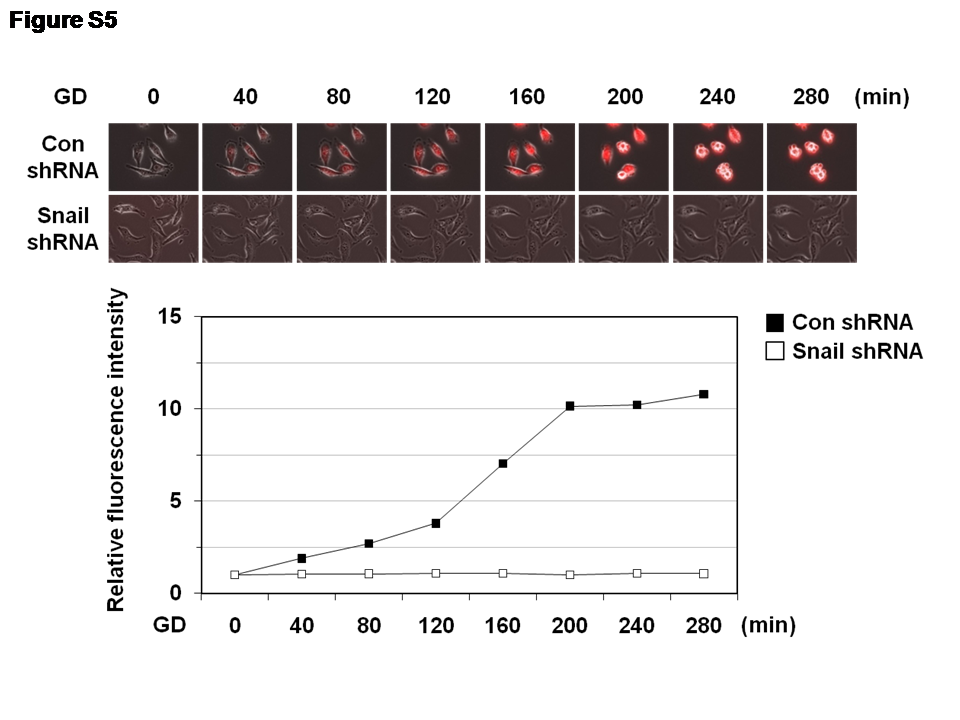

Supplement: Figure S5 — Snail shRNA prevents metabolic stress-induced mitochondrial ROS. MDA-MB-231 control and Snail shRNA stable cells that were treated with 50 nm MitoTracker Red CM-H2XRos were exposed to GD medium and observed by time-lapse microscopy (X400, Carl Zeiss, Axio Observer.D1). Fluorescence images were taken every 5 min for 5 h, and fluorescence intensity was analyzed with Axiovision LE software (Release 4.8 version). Values represent average of total cellular MitoTracker Red CM-H2XRos fluorescence expressed as relative fluorescence intensity at 0 min. (TIF) [file pone.0018000.s005.tif]

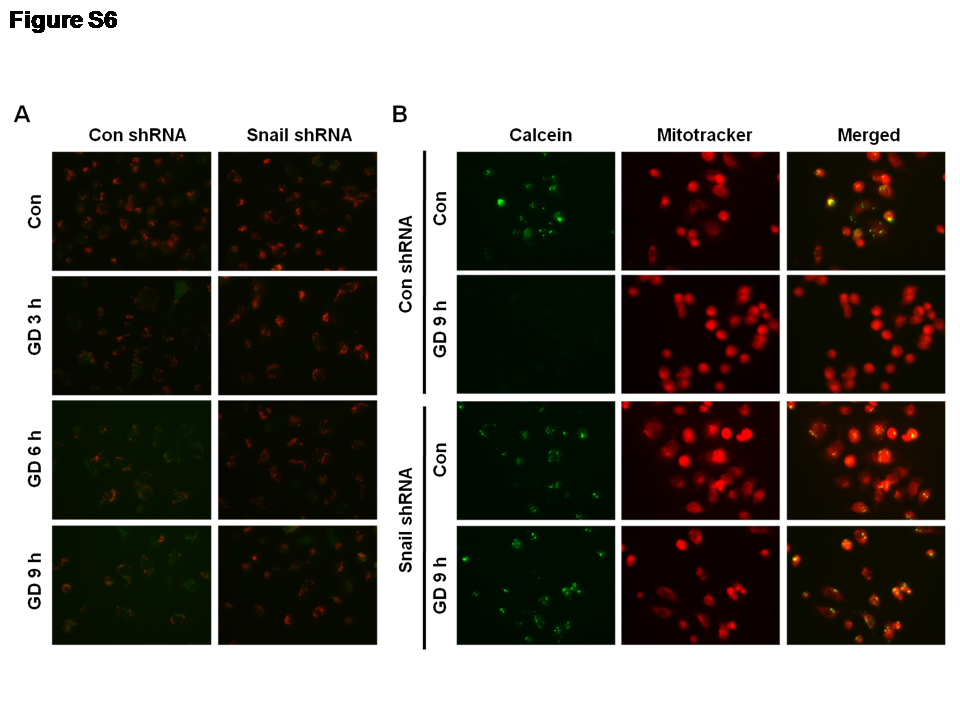

Supplement: Figure S6 — Snail shRNA prevents metabolic stress-induced loss of ΔΨm and mPT. (A) MDA-MB-231 cells stably transfected with control or Snail shRNA were incubated with GD for the indicated times and then treated with 5 mg/ml JC-1 for 15 min, and observed by fluorescence microscopy. (B) MDA-MB231 cells that were transiently transfected with control or Snail shRNA were incubated in normal medium or GD medium for 9 h and loaded with 0.5 µM calcein AM and 5 mM CoCl2 for the final 15 min of the incubation. To detect cytoplasmic mitochondrial distribution, 50 nM MitoTracker CMX-ROS were added during calcein loading. Calcein fluorescence was excited at 488 nm and emitted at 515 nm, MitoTracker Red CMX-ROS was excited at 579 nm, and emitted at 599 nm, and the cells were observed by fluorescence microscopy. (TIF) [file pone.0018000.s006.tif]

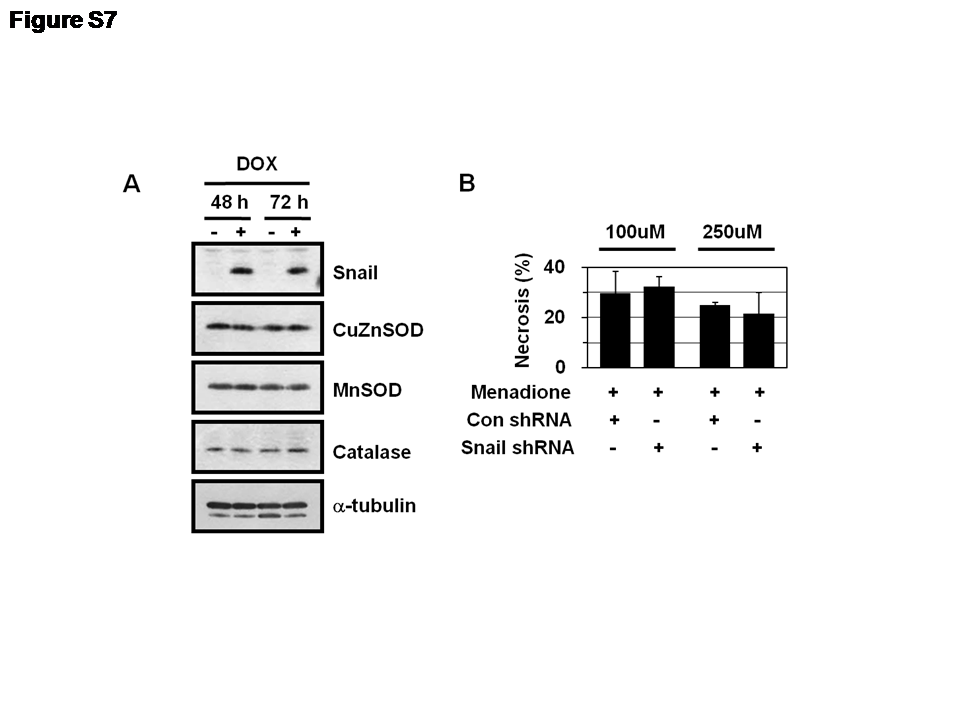

Supplement: Figure S7 — The effects of Snail on antioxidant expression and menadione-induced necrosis. (A) The Snail tet-inducible cell line MCF-7 was treated with DOX for 48 h or 72 h and then analyzed by Western blotting with antibodies against Snail, CuZnSOD, MnSOD, catalase, and α-tubulin. (B) MDA-MB-231 cells that were stably transfected with control and Snail shRNA were cultured in normal growth medium in the presence or absence of 100 µM and 250 µM menadione, stained with HO/PI, and observed under a fluorescence microscope. Results are expressed as mean ± SE from 500 to 800 cells per treatment group and from three independent experiments. (TIF) [file pone.0018000.s007.tif]

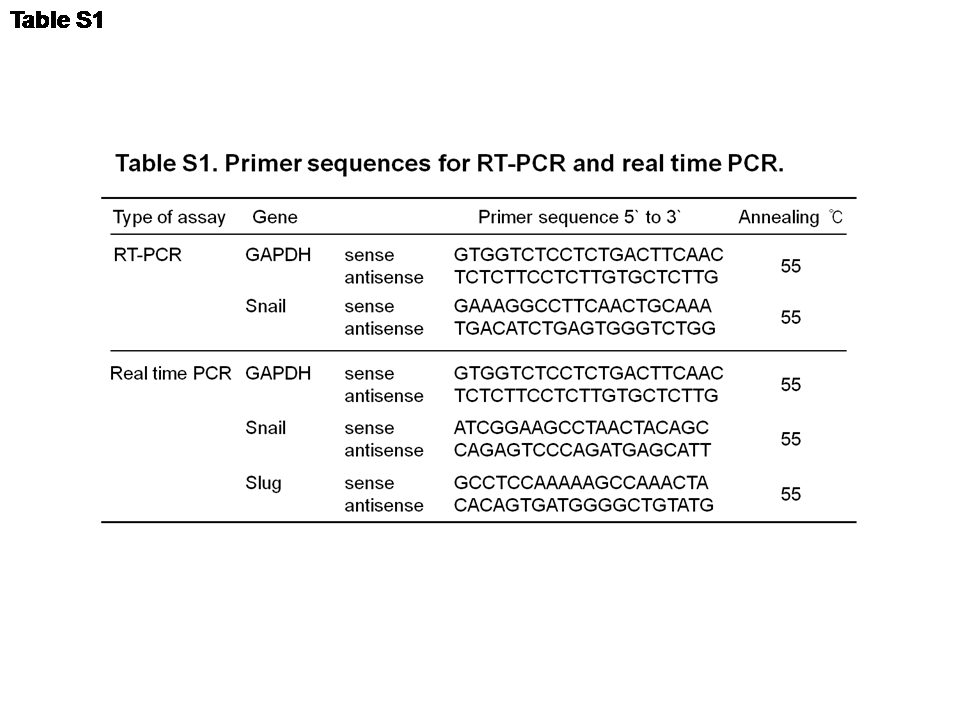

Supplement: Table S1 — Primer sequences for RT-PCR and real time PCR. (TIF) [file pone.0018000.s008.tif]

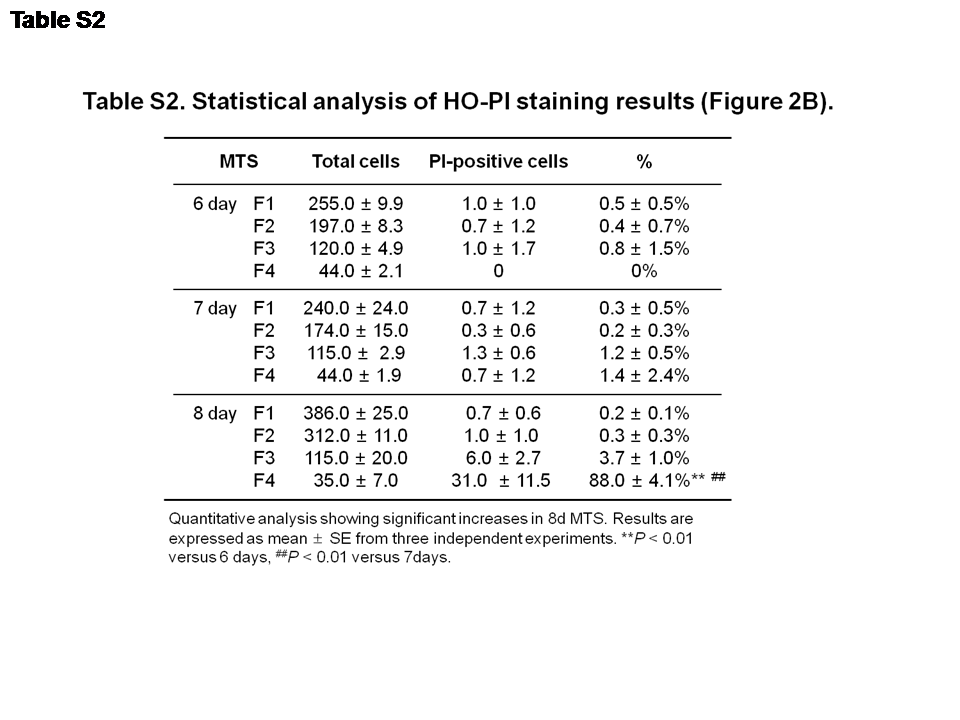

Supplement: Table S2 — Statistical analysis of HO-PI staining results ( Figure 2B ). (TIF) [file pone.0018000.s009.tif]

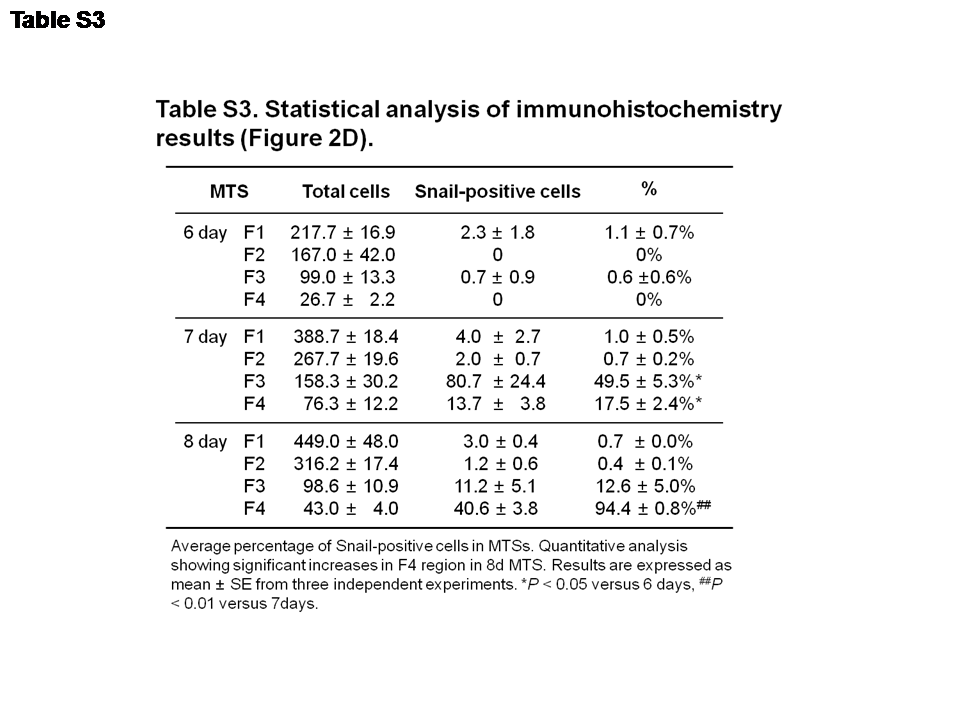

Supplement: Table S3 — Statistical analysis of immunohistochemistry results ( Figure 2D ). (TIF) [file pone.0018000.s010.tif]

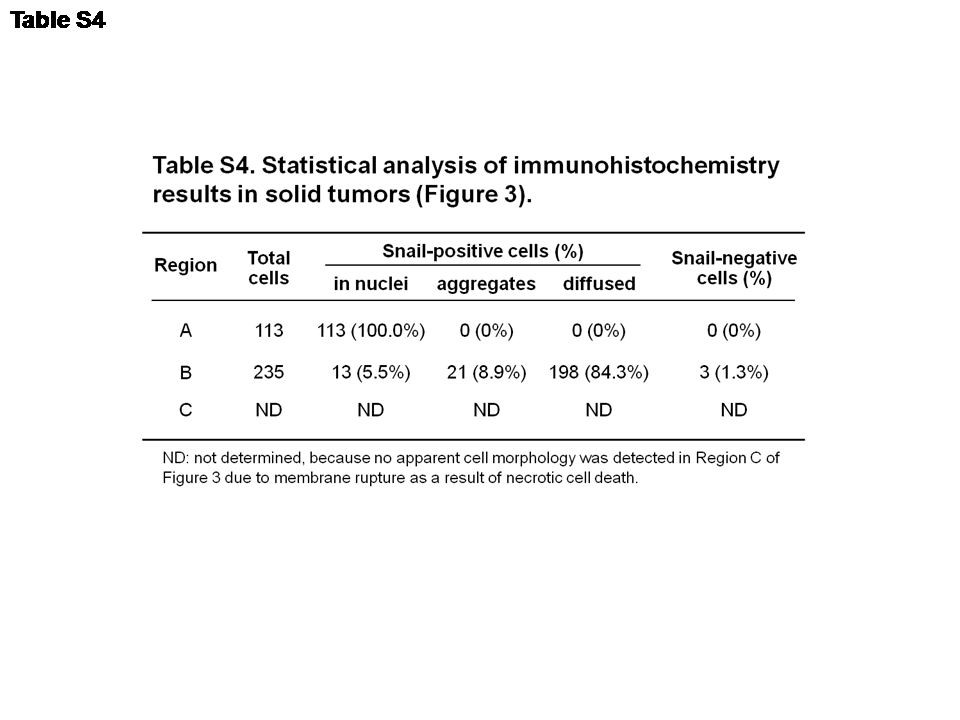

Supplement: Table S4 — Statistical analysis of immunohistochemistry results in solid tumors ( Figure 3 ). (TIF) [file pone.0018000.s011.tif]
